# Evaluation of Inflammatory Markers in Perception Disorders in Major Psychiatric Pathology

**DOI:** 10.3390/ijms26199299

**Published:** 2025-09-23

**Authors:** Laura-Maria Segarceanu, Andrei-Gabriel Zanfir, Dana Galieta Minca, Simona Trifu

**Affiliations:** 1 Department of General Medicine, “Carol Davila” University of Medicine and Pharmacy Bucharest, 020021 Bucharest, Romania; laura.m.segarceanu@stud.umfcd.ro; 2 Doctoral School, “Carol Davila” University of Medicine and Pharmacy Bucharest, 020021 Bucharest, Romania; 3 Department of Complementary Sciences, “Carol Davila” University of Medicine and Pharmacy Bucharest, 020021 Bucharest, Romania; 4 Department of Neurosciences, “Carol Davila” University of Medicine and Pharmacy Bucharest, 020021 Bucharest, Romania; simona.trifu@umfcd.ro

**Keywords:** schizophrenia inflammation, acute psychotic disorder biomarkers, inflammatory markers psychosis, C-reactive protein schizophrenia, cytokines and psychotic disorders, systemic immune–inflammation index, neuroinflammation schizophrenia, psychiatric immunology research

## Abstract

This study investigates the role of inflammatory markers in perception disorders associated with major psychiatric pathology, focusing on schizophrenia and acute psychotic disorder. Guided by the vulnerability–stress–inflammation model, this research explores how genetic predispositions, maternal immune activation, and chronic low-grade neuroinflammation contribute to disease onset and progression. A sample of 135 patients (69 with schizophrenia and 66 with acute psychotic disorder) admitted to a psychiatric hospital between October 2024 and February 2025 was analyzed. Demographic and clinical data, along with biological markers—such as white blood cells, neutrophil-to-lymphocyte ratio (NLR), monocyte-to-lymphocyte ratio (MLR), platelet-to-lymphocyte ratio (PLR), systemic immune–inflammation index (SII), C-reactive protein (CRP), and erythrocyte sedimentation rate (ESR)—were assessed. Results indicated elevated median values for SII, CRP, and MLR, with statistically significant differences compared to normal reference ranges, suggesting persistent systemic inflammation in psychotic disorders. While acute psychotic disorders showed wider value ranges, schizophrenia patients demonstrated higher median levels, consistent with chronic inflammation. No significant differences were observed between the two groups after Bonferroni correction, though CRP values suggested a trend toward greater inflammation in schizophrenia. These findings reinforce the inflammatory hypothesis of psychosis and highlight the potential of biomarkers to refine diagnosis, guide treatment strategies, and support future research into immunomodulatory therapies.

## 1. Introduction

The vulnerability–stress–inflammation model of schizophrenia, discussed by Howes [1] and Muller [2], differs from the traditional vulnerability–stress model of the disease, originally proposed by Zubin and Spring in 1977. Emerging from genetic and epidemiological studies, this model integrates inflammation as a central element, emphasizing the interaction between inflammation and immune dysregulation with genetic and environmental factors.

In the context of this concept, schizophrenia is considered a neurodevelopmental disorder determined by a two-stage (“two-hit”) process. The first stage involves maternal immune activation and increased inflammation during the fetal and perinatal stages. This initial hit “programs” the immune system, particularly affecting the microglia. The second hit usually occurs in late adolescence and triggers a series of changes: dopaminergic dysregulation, increased synaptic elimination, and the onset of psychosis. This phase is characterized by an increase in dopaminergic transmission, which is thought to stem from presynaptic dysfunction and changes in receptor numbers. This hypothesis highlights the essential roles of prenatal and postnatal factors in schizophrenia but does not fully explain the progressive deterioration seen in patients.

Inflammation and stress during critical developmental stages, such as the second trimester of pregnancy or early childhood, are implicated in increasing the risk of schizophrenia. Studies consistently show that stress increases inflammatory markers, reinforcing the link between stress and inflammation.

Genetic factors play a major role, with genetic variants associated with both immune function and susceptibility to schizophrenia. Maternal immune activation during pregnancy can produce epigenetic changes, further influencing disease risk. Large-scale studies have shown that repeated infections and autoimmune diseases increase the likelihood of developing schizophrenia [3], possibly through common genetic pathways.

In a meta-analysis of 145 studies, Rømer et al. [4] observed increased levels of immunological markers in the cerebrospinal fluid of individuals with psychotic disorders compared to healthy individuals.

Numerous other studies confirm the increased presence of inflammatory markers and immune cell activity [5] in people with schizophrenia, both in cerebrospinal fluid and blood.

Genetic studies support the link between polymorphism of genes regulating cytokines IL-6 and IL-10 and predisposition to developing schizophrenia [6]. Immune dysregulation affects neural communication and brain structure in important brain regions.

Recent research suggests that inflammation and immunity may play a role in the etiology of psychotic disorders. Studies conducted on patients experiencing their first psychotic episode, who had not previously been treated with antipsychotics, showed that inflammation is already present at this stage. Some of these abnormalities disappear after treatment is initiated, suggesting that they may be state markers of acute psychosis, but other abnormalities persist. There is evidence to suggest that prenatal infections are involved in the etiology of schizophrenia. Several hypotheses link inflammation and immunity to psychotic disorders [7].

Maternal immune activation (MIA) during pregnancy has been associated with an increased risk of schizophrenia in offspring. Epidemiological studies, including a recent meta-analysis [8] that included 23 observational studies, show that prenatal infections may increase the risk of psychosis, with odds ratios ranging from 1.25 to 2–5 times higher.

It is assumed that inflammatory molecules, such as cytokines and chemokines (TNF-α, IL-1β, IL-6), triggered by prenatal exposure to pathogens, can cross the placenta. These affect fetal brain development and cause lasting disruptions in neurodevelopment. Animal studies support this theory, highlighting behavioral abnormalities, cortical thinning, disturbances in the development of cortical GABAergic neurons, and disruptions in brain connectivity—features also observed in schizophrenia.

Microglia are the most important component of the local immune system in the central nervous system (CNS) and constitute between 10% and 20% of all cells in the CNS. They play an essential role in neuroinflammation and provide the first line of defense in the event of injury or disease [9].

In schizophrenia, these cells can become activated or “sensitized” by stimuli such as aging, neurodegeneration, or stress, leading to an exaggerated inflammatory response. Once sensitized, microglia can respond to additional low-intensity stimuli (minor systemic inflammation) by proliferating and increasing the production of proinflammatory cytokines [10].

According to studies, this chronic low-intensity neuroinflammation is associated with structural changes in the brain, including reduced gray and white matter volume, and is linked to the onset of schizophrenia symptoms [11].

Recent research has consistently identified elevated levels of proinflammatory cytokines in individuals with schizophrenia and acute psychosis. Interleukin-6 (IL-6), tumor necrosis factor alpha (TNF-α), interleukin-1 beta (IL-1β), and interferon gamma (IFN-γ) have been found in high concentrations in the serum, cerebrospinal fluid, and brain tissue of patients, particularly during acute episodes. These elevated cytokines are associated with the severity of psychotic symptoms, cognitive deficits, and treatment resistance. Meta-analyses and more recent research also report elevated levels of anti-inflammatory cytokines, including TGF-β1, IL-1RA, and IL-4, in patients with longer disease duration or chronic schizophrenia spectrum disorders. Although there is evidence of increased levels of anti-inflammatory cytokines in schizophrenia, further research is needed to clarify their role and reliability as clinical markers [12].

Previous studies have shown that there is an interaction between oxidative stress and neuroinflammation [13]. Chronic stress and inflammation can activate the NF-κB pathway, which stimulates the production of prostaglandins and reactive oxygen species. These factors can interact, amplifying each other and increasing inflammation, which contributes to the onset of psychotic symptoms.

It has been observed that certain interleukins appear to significantly affect neurotransmitter systems in ways that may contribute to the onset of schizophrenia—IL-1β promotes the development of dopaminergic neurons, while IL-6 decreases the survival of serotonergic neurons. Activation of the immune system during pregnancy can lead to an increase in the number of dopaminergic neurons in the fetal brain, which could cause dopamine imbalances associated with schizophrenia [2]. Animal studies [14] have shown that chronic inflammation leads to decreased dopamine release in the striatum, resulting in anhedonia. This is a characteristic negative symptom, particularly present in chronic schizophrenia.

Glutamate is the most abundant neurotransmitter in the brain and is considered a key factor in the pathogenesis of schizophrenia, particularly through its involvement in tryptophan/kynurenine metabolism, mediated by NMDA receptors. The proposed mechanism in schizophrenia involves a predominant type II immune response, resulting in increased production of kynurenic acid, a natural antagonist of NMDA receptors. This inhibits glutamate transmission—a possible mechanism in the onset of schizophrenia symptoms. Antibodies against NMDA receptors were found in approximately 10% of untreated patients, supporting this hypothesis [15].

According to studies, postmortem findings have identified fibrin degradation products in the brain and cerebrospinal fluid (CSF) of approximately 50% of patients with schizophrenia. Microglia density is significantly increased in schizophrenia, particularly in the temporal cortex [16].

Among the categories of inflammatory markers associated with schizophrenia and acute psychosis are cytokines [17], chemokines [18], acute-phase proteins, and hematological markers.

A meta-analysis found higher levels of proinflammatory cytokines in peripheral blood in both first-episode and relapse patients compared to healthy individuals. However, higher levels of anti-inflammatory cytokines were also observed in these patients compared to the control group [19]. The cytokine imbalance depends on the phase of the disease. Patients experiencing their first acute psychotic episode showed significant increases in interferon-γ (IFN-γ), IL-1RA, IL-1β, IL-6, IL-8, IL-10, IL-12, sIL-2R, TGF-β, and TNF, while IL-4 levels were significantly decreased. Age, sex, disease duration, smoking, and body mass index (BMI) were not associated with increases in IL-6 and TNF-α in acute psychosis. During acute exacerbation of schizophrenia, increases in IFN-γ, IL-1RA, IL-1β, IL-6, IL-8, IL-12, sIL-2R, TGF-β, and TNF were observed, along with decreases in IL-4 and IL-10 levels, compared to the control group. In patients with schizophrenia outside the acute episode, IL-6, TNF, sIL-2R, and IL-1β levels were elevated, and IFN-γ levels were decreased compared to those of healthy individuals. No significant differences were found in IL-2, IL-4, or IL-10 levels. Age, sex, disease duration, smoking, and body mass index (BMI) were not associated with the association between IL-6 and schizophrenia. Similarly, a meta-analysis of cytokines in the cerebrospinal fluid (CSF) of patients with schizophrenia showed increased levels of IL-6 and IL-8. In conclusion, IL-6 is the cytokine whose increase is most consistently observed in all phases of schizophrenia. A large number of other cytokines are also affected [19].

Changes in chemokines are another example of an aberrant immune response in patients with schizophrenia. Elevated levels of MCP-1, IL-8, eotaxin-1, MIP-1α, and MIP-1β have been identified. According to a meta-analysis, MCP-1 is the only chemokine that is elevated in both acute psychosis and schizophrenia, suggesting that it may be a stable marker, present from the onset and persistent throughout the course of the disease [18].

Recent studies have identified several acute-phase proteins that are significantly altered in schizophrenia and acute psychosis, indicating the presence of an inflammatory component in these disorders. C-reactive protein (CRP) is a major marker of inflammation, synthesized by the liver in response to cytokine stimulation [15]. A study evaluating 174 individuals with acute psychosis found that 59.8% of them had elevated CRP levels, which remained high throughout their hospitalizations. Among patients with schizophrenia, 43% had elevated CRP levels, compared with 20% among healthy individuals in the control group. The study also reported that elevated CRP levels were associated with poorer cognitive performance and reduced cortical thickness [20]. Other acute-phase proteins that have been found to be significantly elevated in schizophrenia are fibrinogen, haptoglobin, complement components C3 and C4, alpha-1-acid glycoprotein, and hemopexin [21].

Complete blood counts are often used in psychiatric patients to assess general health or to monitor changes in blood parameters related to drug treatment [22]. Schizophrenia spectrum disorders are associated with immunoinflammatory activation. Inflammation indices based on complete blood counts, such as the neutrophil-to-lymphocyte ratio (NLR), monocyte-to-lymphocyte ratio (MLR), and platelet-to-lymphocyte ratio (PLR), are reproducible and cost-effective markers of inflammation in mental disorders [23].

Some studies have suggested that inflammatory indices may be biomarkers of acute exacerbations in schizophrenia. In a 2024 cohort study, Zheng et al. showed that NLR was significantly higher in the acute phase than in the stable phase of schizophrenia and that this elevation was independent of antipsychotic exposure, highlighting NLR’s potential as an objective indicator of clinical decompensation [24]. Complementing this, Sugita et al. retrospectively analyzed 251 inpatients and reported that higher admission NLR tracked acute symptom severity and that within-episode changes in NLR paralleled treatment response, supporting its use for short-term monitoring in the hospital setting [25]. Beyond single cohorts, a narrative review from 2024 also appraised NLR as an “emerging” inflammatory marker in psychotic disorders, while emphasizing the need to account for confounders (infection, smoking, BMI) when interpreting values [26]. Other readily derived indices—monocyte-to-lymphocyte (MLR) and platelet-to-lymphocyte ratios (PLR)—appear to move with acute symptom fluctuation as well. In a relapse–remission comparison of 105 patients, it has been found that NLR, MLR, and PLR were all significantly elevated during psychotic relapse versus remission and healthy controls, suggesting that a CBC-based panel can capture the inflammatory surge that accompanies exacerbations [27].

Extending this vision, studies evaluating the systemic immune–inflammation index (SII = platelets×neutrophils/lymphocytes) reported higher SII among patients hospitalized for schizophrenia exacerbation; in one inpatient series, SII, NLR, MLR, and PLR collectively discriminated acutely ill cases, underscoring their utility as inexpensive adjuncts to clinical assessment [28]. PMC Broader cross-sectional work also shows that schizophrenia patients have higher neutrophils, monocytes, platelets, and the ratios NLR, PLR, and MLR than controls, reinforcing a disorder-related inflammatory profile onto which state changes (exacerbations) are superimposed [29].

Hematological markers that show changes in schizophrenia and acute psychotic disorder are as follows:

- Leukocyte formula: increased levels of total leukocytes, granulocytes, and monocytes have been identified in patients experiencing their first psychotic episode, compared to healthy individuals [19]. According to another study, these markers decrease significantly after 4 weeks of antipsychotic treatment. This suggests a reduction in systemic inflammation as clinical status improves [30].
- Neutrophil-to-lymphocyte ratio (NLR) and monocyte-to-lymphocyte ratio (MLR): Research comparing acute psychotic disorder and substance-induced psychosis has indicated higher levels of NLL and MLL in both groups compared to control groups, suggesting that these ratios may be potential markers of inflammatory processes in acute psychosis [31].
- Platelet–lymphocyte ratio (PLR): Increased PLR has been associated with treatment resistance in schizophrenia, making it a potential marker for treatment response [32].
- Erythrocyte sedimentation rate (ESR): This was found to be elevated in patients experiencing their first psychotic episode. ESR levels remained unchanged in a significant proportion of patients after 4 weeks of treatment with antipsychotics. Non-responders had higher ESR levels compared to those who responded to treatment. Therefore, persistently elevated ESR may indicate a poor response to treatment [30].
- Systemic inflammation index (SII): This is a marker that reflects immune response and inflammation, defined as follows: SII = Platelets x Neutrophils/Lymphocytes. The increased SII value in patients with schizophrenia compared to healthy individuals suggests its potential as a diagnostic marker for identifying inflammatory changes during psychotic episodes [33].

## 2. Results

### Demographic and Clinical Features

The study was conducted on a sample of 135 subjects with psychosis, of whom 69 had schizophrenia (51.11%) and 66 had acute psychotic disorder (48.89%). Of these, 127 are male (94.07%) and only 8 are female (5.93%), 113 come from urban areas (83.7%) and 22 from rural areas (16.3%). The patients’ ages are distributed approximately normally, ranging from 18 to 64 years, with a mean of 39.5 years and a standard deviation (SD) of 11.09. In terms of education level, the majority of 79 patients (58.52%) have between 9 and 12 years of schooling, 29 have >12 years of schooling (21.48%), and 27 have <9 years (20%). In terms of relationship status, 82 do not have a stable partner (64.45%), and 48 have a stable partner (35.55%).

The descriptive analysis for the numerical variables at the level of the entire batch is illustrated in Table 1. It contains the mean ± standard deviation, minimum, maximum, and skewness coefficient for normally distributed variables, and the median with interquartile range [Q1–Q3], minimum, maximum, and skewness coefficient for asymmetrically distributed variables. The variables Age, Lymphocytes, and RPL can be considered to be approximately normally distributed, given the visual inspection of the graphs, which are relatively symmetrical, and the skewness coefficient, which has a value between −1 and 1.

It can be observed that the median values for NRL, MRL, SII, and CRP are slightly elevated compared to the upper limit of the normal reference range (2, 0.25, 400, 1 mg/L), suggesting a possible pattern of more pronounced inflammation in patients with schizophrenia and acute psychosis—an aspect also noted in the literature. However, we cannot make a generalization at this stage, as the hypothesis needs to be explored through statistical significance tests.

**Figure 1:**
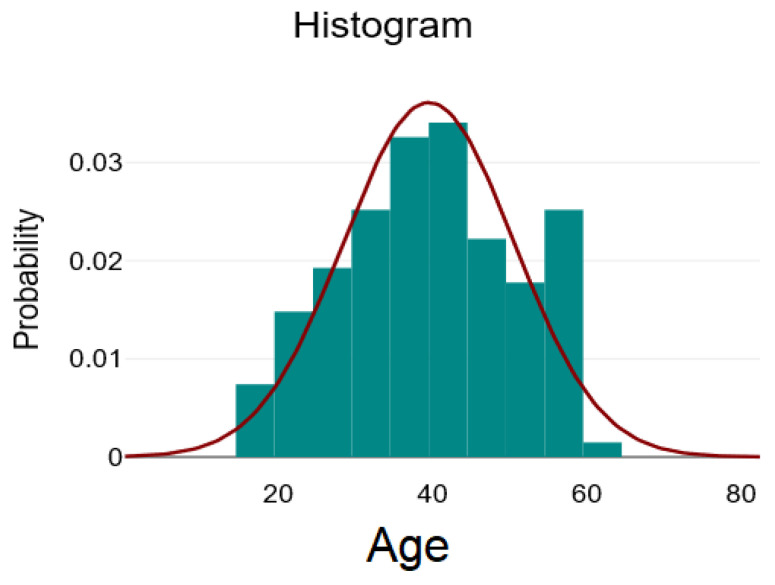
Distribution of the age variable—green bars represent the histogram of observed age values (frequency counts), the red line depicts the fitted normal distribution curve, showing how closely the data follows a normal distribution.

**Figure 2:**
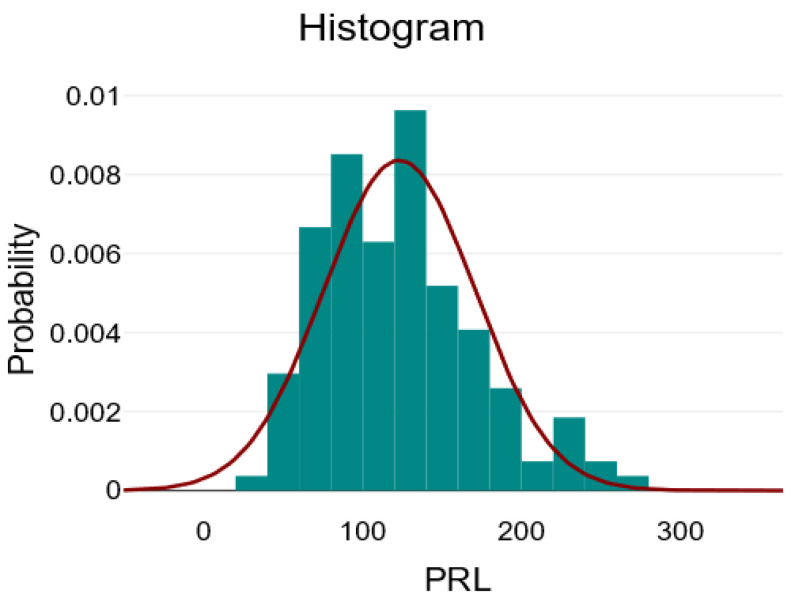
Distribution of the blood platelet/lymphocyte ratio (PRL) variable—green bars represent the histogram of observed PRL values (frequency counts), the red line depicts the fitted normal distribution curve, showing how closely the data follows a normal distribution.

**Figure 3:**
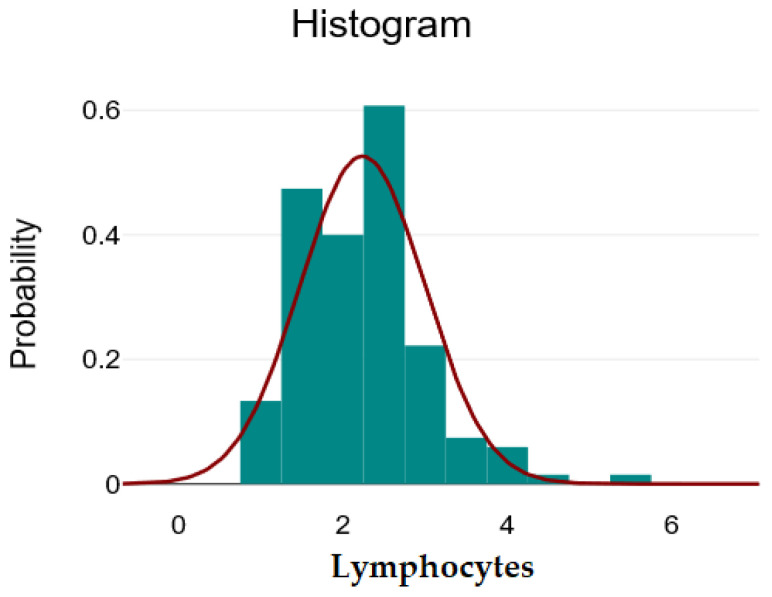
Distribution of the lymphocytes variable—green bars represent the histogram of observed Lymphocytes values (frequency counts), the red line depicts the fitted normal distribution curve, showing how closely the data follows a normal distribution.

In Figure 1, Figure 2 and Figure 3, we present the histograms showing the relatively normal distribution of data for the variables: age, lymphocytes, and PRL.

**Figure 4:**
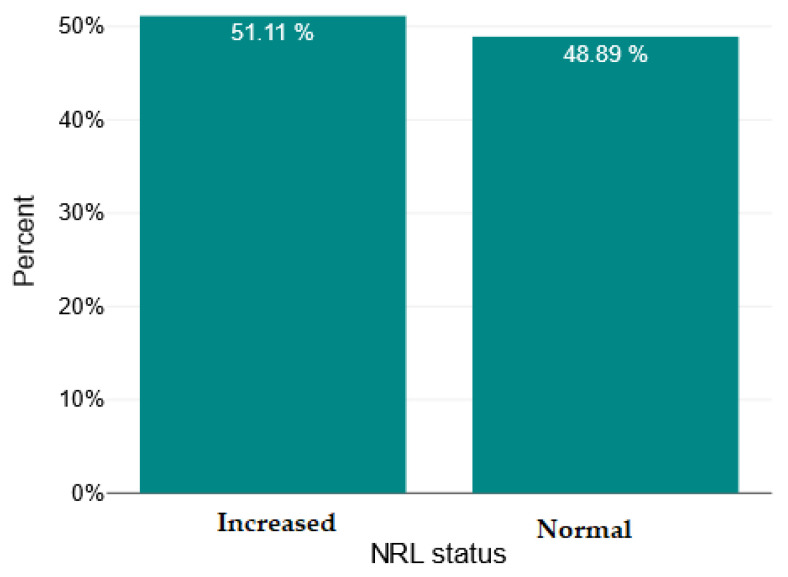
Normal versus increased neutrophil/lymphocyte ratio (NRL).

**Figure 5:**
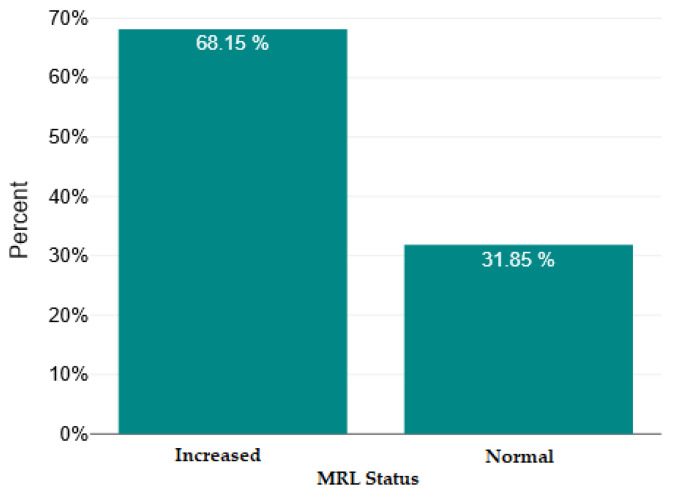
Normal versus increased monocyte/lymphocyte ratio (MRL).

**Figure 6:**
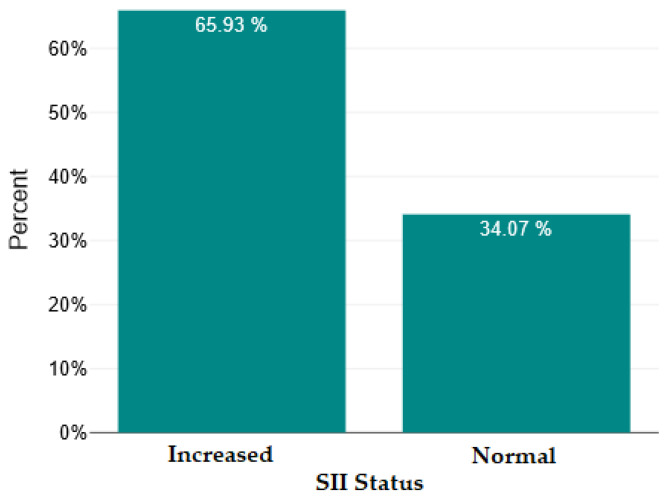
Normal versus increased systemic immune–inflammatory index (SII).

**Figure 7:**
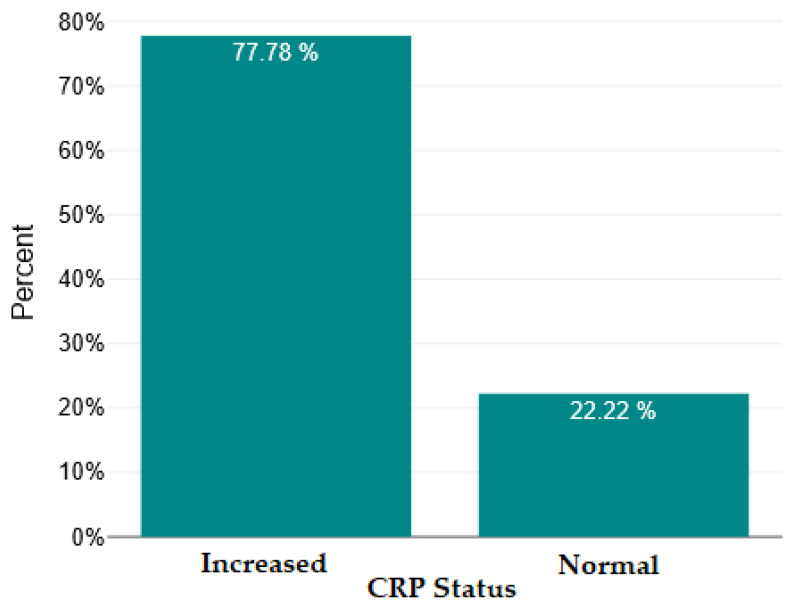
Normal versus increased C-reactive protein (CRP).

In Figure 4, Figure 5, Figure 6 and Figure 7, we show the bar charts that visually illustrate the percentage of patients with elevated RNL, RML, SII, and PCR values, respectively, compared to the percentage of patients with normal values (below the upper limit), according to the clinical reference values mentioned in the Section 4.

**Figure 8:**
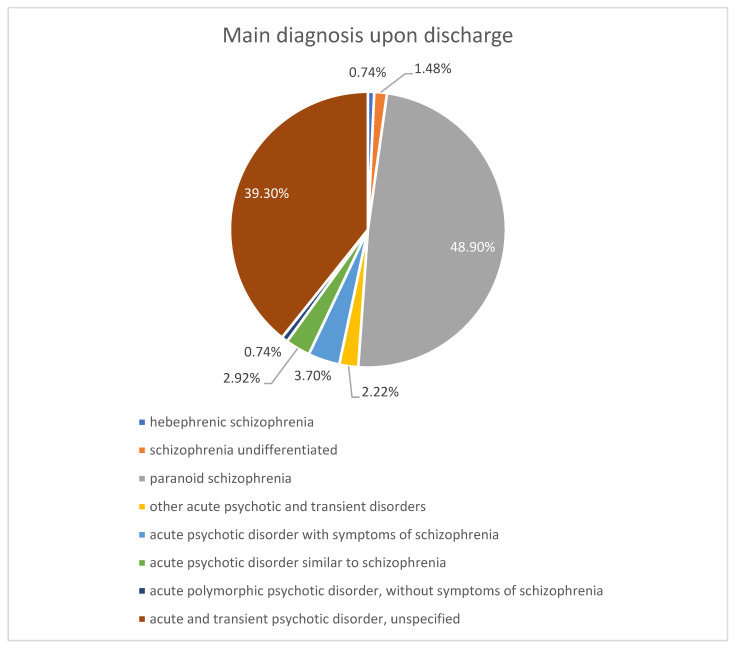
Distribution by diagnosis.

From a diagnostic point of view, most patients in the schizophrenia group have a diagnosis of paranoid schizophrenia, and most patients with acute psychotic disorder have a diagnosis of acute and transient psychotic disorder, unspecified. These proportions reflect the normal distribution observed in clinical practice. Figure 8 shows the exact diagnoses and their proportion in the study group.

Categorical and continuous demographic variables according to the diagnostic group are presented in Table 2. For age, we calculated the mean and standard deviation, as it is normally distributed in both groups. For the variables of gender, background, level of education, and relationship status, we specified the number and percentage of patients.

Comparing the nominal variables between the two diagnostic groups, we observed that there were no significant differences in gender distribution (Fisher’s exact test, *p* = 0.275), background (chi-square test, *p* = 0.13), level of education (chi-square test, *p* = 0.635), and relationship status (chi-square test, *p* = 0.103). Therefore, these were not included as covariates in subsequent analyses.

**Figure 9:**
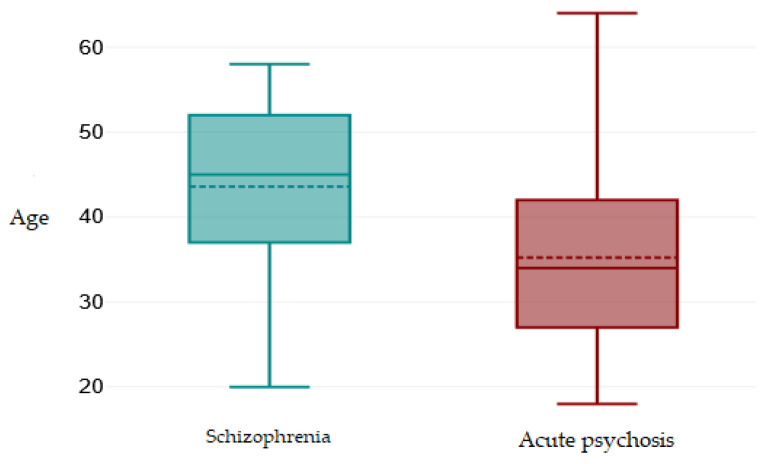
Age distribution according to diagnostic group.

Given the normal distribution of age in both groups, we compared the two groups using the *t*-test for independent samples. Patients with acute psychotic disorder are significantly younger than those with schizophrenia (*p* = <0.001, 95% confidence interval [4.83–11.87]), reflecting the normal pattern of presentation of the first psychotic episode at younger ages. Figure 9 visually illustrates this difference.

Descriptive statistics for comparing inflammatory markers between the two groups are shown in Table 3. We specified the asymmetry coefficient to provide additional context regarding the shape of each variable’s distribution in order to choose the most appropriate measure of central tendency and dispersion (mean/median; SD/IQR). Since only the RPL variable has an approximately normal distribution in both groups, we reported the mean and standard deviation only in this case. In situations where a variable has a normal distribution in only one of the groups, we reported the median and interquartile range for both groups to maintain statistical consistency. The minimum and maximum were reported to highlight the full range of data variation.

We can observe that for almost all parameters studied (except lymphocytes), the widest range of values is found in the acute psychosis group, as well as the highest maximum value. However, for each inflammatory marker, the highest median value (i.e., mean for RPL) is found in the schizophrenia group, suggesting a potential tendency toward a more pronounced inflammatory profile in patients with schizophrenia compared to those with acute psychosis. This is a finding based on descriptive analysis, so it needs to be confirmed by inferential statistical tests.

We attempted to determine whether there were (alternative hypothesis) or were not (null hypothesis) statistically significant differences between the means/medians of inflammatory markers in the study sample and the upper limits of normal clinical ranges.

Since in the descriptive analysis we observed medians/means higher than the corresponding upper normal limits only for the NRL, MRL, SII, and CRP markers, only these were tested for significance. To assess whether the medians of these markers in the study group are significantly increased compared to the upper reference values, we used Wilcoxon tests for a single sample (“one-sample Wilcoxon signed-rank tests”), with a unidirectional alternative hypothesis (median > upper normal value).

To control for a potential type I error generated by performing four Wilcoxon tests, we applied the Bonferroni correction—a conservative method of adjusting the statistical significance threshold when performing multiple tests. The significance threshold was thus adjusted to 0.0125 (0.05/4). Results with *p* < 0.0125 were considered significant.

Table 4 presents the results of the Wilcoxon tests for a single sample for markers with increased median value at a descriptive level and with asymmetric distribution (NRL, SII, CRP, MRL), which justifies the use of the nonparametric test.

Wilcoxon tests with a theoretical mean equal to the upper normal limit showed that the median values of the SII, CRP, and MRL markers are significantly higher than the reference values (*p* < 0.0125). The statistical difference is very significant, even after Bonferroni correction for multiple tests, given the *p*-value < 0.001; the median value of NRL is not significantly higher than the upper reference value (*p* = 0.089).

Therefore, the null hypothesis is rejected for SII, CRP, and MRL, and the null hypothesis cannot be rejected for NRL.

**Figure 10:**
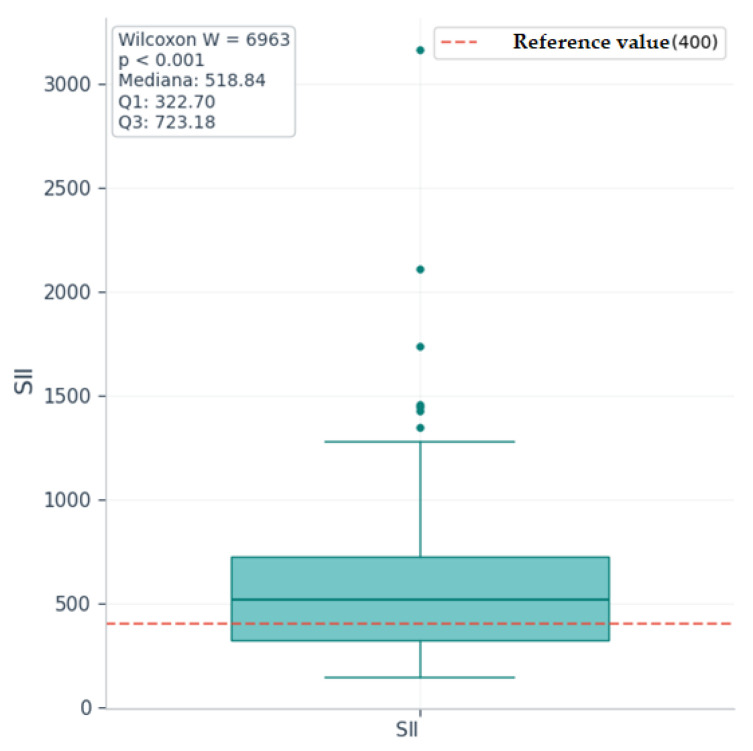
Boxplot for systemic immune–inflammatory index (SII) values with Wilcoxon test results.

**Figure 11:**
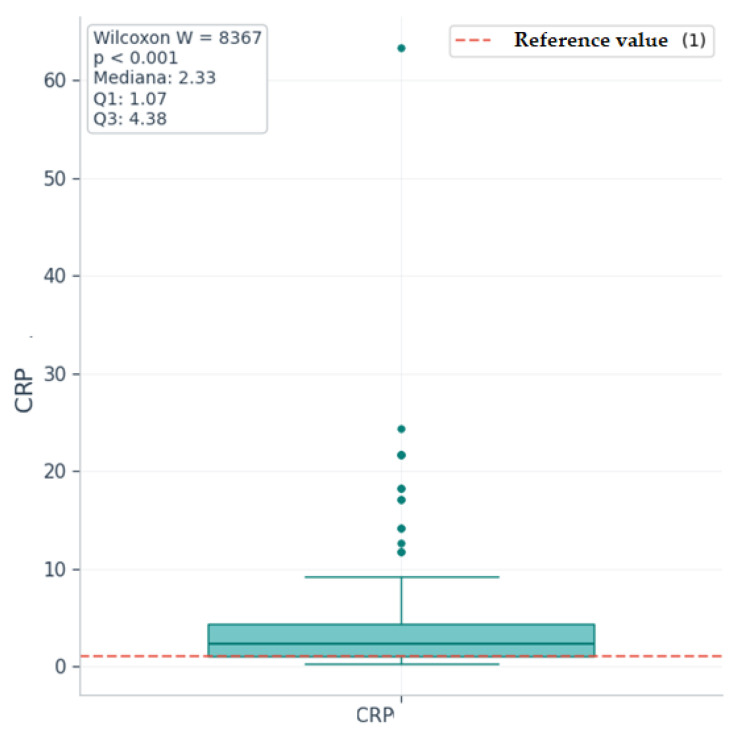
Boxplot for C-reactive protein (CRP) values with Wilcoxon test results.

**Figure 12:**
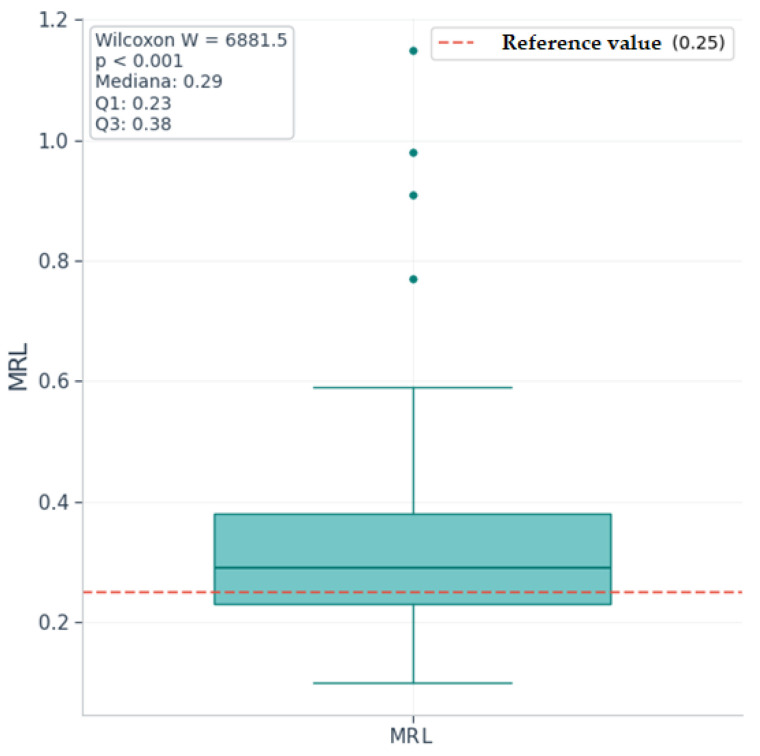
Boxplot for monocyte/lymphocyte ratio (MRL) values with Wilcoxon test results.

Figure 10, Figure 11 and Figure 12 are boxplot graphs that include the distribution of values at the sample level (with percentiles 25, 50, 75, and extreme values), the reference value used for comparison, medians, quartiles Q1 and Q3, and the results of the Wilcoxon tests (W and *p*).

Calculating the effect size r for each marker using the formula (effect classification according to Cohen [34]: very small < 0.10, small < 0.3, medium ≈ 0.3–0.5, large > 0.5), we obtain a medium to large effect size for SII and MRL (r = 0.45) and a large effect size for CRP (r = 0.71), suggesting clinically relevant differences.

We assume the existence (alternative hypothesis) or non-existence (null hypothesis) of statistically significant differences between the values of inflammatory markers in the group of patients with schizophrenia and those in the group of patients with acute psychotic disorder.

Most of the recorded markers have an asymmetric distribution in both diagnostic groups, in which case we compared the median values between the two groups using nonparametric Mann–Whitney U tests. For the PRL variable, we compared the mean values between the two groups using the *t*-test for independent samples, as it has a normal distribution in both groups, and the Levene test confirmed the absence of significant differences (*p* > 0.05) between variances. The tests were performed bilaterally (two-tailed), assuming the possibility of differences in both directions.

To control the risk of type I errors associated with performing ten tests (nine Mann–Whitney and one *t*-test), we applied the Bonferroni correction. The adjusted significance threshold was 0.005 (0.05/10). Thus, *p*-values < 0.005 were considered significant.

Table 5 presents the results of the Mann–Whitney U tests for comparing the markers: leukocytes, neutrophils, lymphocytes, monocytes, NRL, MRL, SII, CRP, and ESR between the two diagnostic groups. Table 6 presents the result of the *t*-test for independent samples for comparing the PRL variable between the two groups.

The results of the Mann–Whitney U and t tests for independent samples showed that, for the variables leukocytes, neutrophils, SII, and ESR, there are slightly higher median values in patients with schizophrenia than in those with acute psychotic disorder, but the difference is not statistically significant (neither at the 0.05 significance threshold nor at the corrected threshold), and the effect size is small (<0.3), so the null hypothesis cannot be rejected; similarly, for the variables lymphocytes, monocytes, NRL, and MRL (higher values in patients with schizophrenia, statistically insignificant differences), but the effect size is very small (<0.10), so the null hypothesis cannot be rejected. For the CRP variable, there are slightly higher median values in patients with schizophrenia compared to those with acute psychotic disorder, but the difference did not reach statistical significance after applying the Bonferroni correction for multiple tests; given that the correction method used is conservative and reduces the risk of type I errors, but may increase the risk of type II errors (failure to identify real differences), and the difference is significant according to the standard threshold of 0.05, the result should be interpreted with caution. For the PRL variable, the difference between the schizophrenia group and the acute psychotic disorder group is not statistically significant, the effect size is very small (<0.10), and the null hypothesis cannot be rejected.

**Figure 13:**
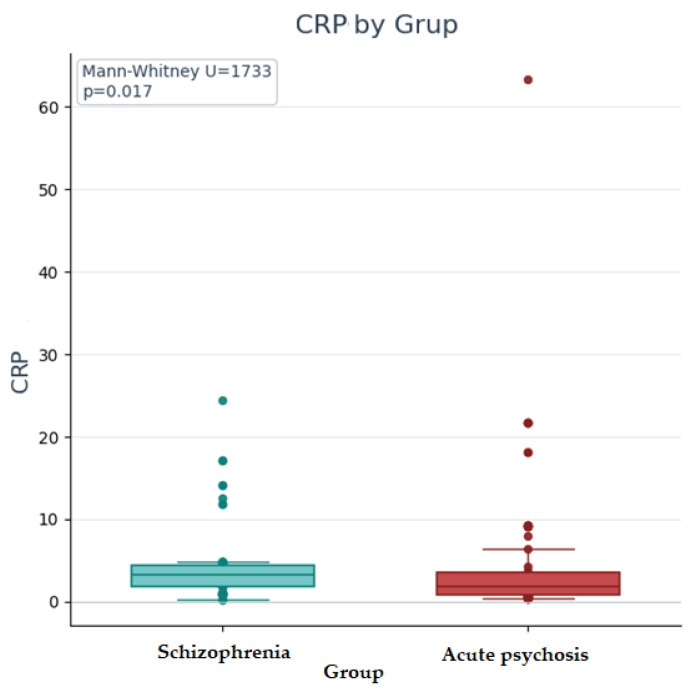
Boxplot with C-reactive protein (CRP) values by group, with Mann–Whitney U test results.

Figure 13 shows the boxplot graph for CRP values according to diagnostic group, with the results of the Mann–Whitney U test (U, *p*). It can be seen that CRP values have a higher median in the schizophrenia group compared to the acute psychosis group. Although the boxplot suggests a tendency for a difference in the central position of CRP values, this was not supported by statistical significance after applying the Bonferroni correction.

## 3. Discussion

The study presents both the specific limitations of observational, retrospective, cross-sectional, and comparative designs, as well as certain particular limitations related to the context and methodology applied. These include the inability to establish causal relationships due to the observational and cross-sectional nature of the study; lack of control over data collection, as the study is retrospective; selection bias—we only selected patients who had laboratory tests available at the time; analysis at a single point in time (cross-sectional) did not allow for a dynamic assessment of inflammatory markers; lack of standardized reference values for derived indices (NRL, MRL, PRL, SII) made it difficult to evaluate these markers and identify values that could suggest the presence of inflammation; most patients hospitalized with schizophrenia were in a phase of acute exacerbation of the disease, a situation in which additional inflammatory changes may occur in addition to the chronic inflammation specific to the disease—this aspect may affect the homogeneity of the group and the consistency of the results; and the sample size (135 patients) may limit the statistical power and generalizability of the results.

## 4. Materials and Methods

Chronic neuroinflammation associated with schizophrenia may imply a higher baseline level of inflammatory markers in these patients compared to healthy individuals, and the increase in values during acute episodes correlates with symptom severity and treatment resistance. Numerous inflammation biomarkers may be elevated in patients with acute psychosis, both in the diagnosis of schizophrenia and in transient forms. We have not identified any studies in the literature that present the possible differences between these two diagnostic groups.

The importance of the study lies in the clinical implications of confirming the hypotheses: the potential of the evaluated markers to guide therapeutic decisions; the exploration of immunomodulatory therapies as adjuvants to antipsychotics in certain cases; and the possible identification of types of psychotic disorders that associate a significant inflammatory component.

Starting from the objectives of the study that include assessment of systemic inflammation in patients with perception disorders caused by psychiatric pathology and identification of differences between diagnostic subgroups of patients in terms of the inflammatory markers studied we highlighted some working hypotheses: the null hypothesis: the mean/median values of the inflammatory markers studied in patients with psychosis are not statistically significantly higher than the upper limits of the normal reference ranges used in medical practice; alternative hypothesis: the mean/median values of the inflammatory markers studied in patients with psychosis are statistically significantly higher than the upper limits of the normal reference ranges used in medical practice and the null hypothesis: there are no statistically significant differences between the mean/median values of the markers studied in patients diagnosed with acute psychotic disorder and those diagnosed with schizophrenia with alternative hypothesis: there are statistically significant differences between the mean/median values of the markers studied in patients diagnosed with acute psychotic disorder and those diagnosed with schizophrenia.

The study was conducted on a sample of 135 patients admitted to a Clinical Psychiatric Hospital between 1 October 2024 and 1 February 2025, aged between 18 and 64, male and female, diagnosed with schizophrenia or acute psychotic disorder, based on the diagnostic criteria of DSM-5 and ICD-10. The inclusion criteria for the study were diagnosis at discharge of schizophrenia—in all clinical forms described by ICD-10—and acute and transient psychotic disorder—described in ICD-10; age between 18 and 65; availability of blood tests, including the markers investigated. The exclusion criteria were diagnosis of acute psychotic disorder attributed to substance use, as the etiological mechanisms and clinical manifestations of these episodes differ significantly from those of primary psychotic disorders; also, psychoactive substances can directly influence the biological markers analyzed, affecting the validity of the results; age over 65 years, to reduce the biological and clinical variability associated with comorbidities and age-related physiological changes that could influence the interpretation of the results regarding inflammatory markers; the presence of concomitant marked affective symptoms, to maintain the homogeneity of the sample and the internal validity of the study [35].

The factual variables described were age, gender, background, level of education, and relationship status. The biological variables that objectify systemic inflammation were white blood cell count, neutrophil count, lymphocyte count, monocyte count, neutrophil/lymphocyte ratio (NLR), monocyte/lymphocyte ratio (MLR), platelet/lymphocyte ratio (PLR), systemic immune–inflammatory index (SII), C-reactive protein (CRP), and erythrocyte sedimentation rate (ESR).

In order to simplify the subsequent statistical analysis, certain variables were redefined as follows: the level of schooling was operationalized as a categorical variable with three levels: <9 years of schooling (those who mentioned in the clinical interview that they had never started high school), 9–12 years of schooling (those who mentioned in the clinical interview that they dropped out of high school, finished high school or vocational school, or are still in high school), >12 years of schooling (those with higher education or post-secondary education); relationship status was operationalized as a binary variable: no stable partner (answers such as “single” and “divorced”), with a stable partner (“married,” “cohabiting,” and “in a relationship”); biological variables were analyzed both as a continuous variable (exact value) and categorically (normal vs. elevated), based on standard reference ranges provided by the laboratory and the literature for derived indices (NRL [36], MRL [37], PRL [38], SII [39])—according to studies on healthy populations or populations with schizophrenia that specify similar thresholds. Thus, in order to highlight a potential low-grade chronic inflammation specific to schizophrenia, we defined the values of the inflammatory markers studied as elevated as follows: Leukocyte count > 11 × 103/µL, Neutrophil count > 8 × 103/µL, Lymphocyte count > 4.5 × 103/µL, Monocyte count > 1 × 103/µL, NRL (calculated as neutrophil count/lymphocyte count) > 2.0, MRL (monocyte count/lymphocyte count) > 0.25, PRL (platelet count/lymphocyte count) > 150, SII (number of blood platelets × number of neutrophils/number of lymphocytes) > 400, CRP > 1 mg/L and ESR > 15 mm/h. The values mentioned correspond to the clinical reference thresholds above which the existence of at least low-intensity inflammation can be considered.

The data were initially processed in Microsoft Excel, and statistical analysis was performed using the DATAtab online platform (www.datatab.net accessed on 5 March 2025). Descriptive statistics were used to characterize the study population from a demographic and clinical perspective. For numerical variables, the following were specified: mean, standard deviation (SD), minimum, maximum, and asymmetry coefficient for approximately normal distributions; median, interquartile range (IQR), minimum, maximum, and asymmetry coefficient for nonparametric variables.

The normality of the distribution was assessed based on: normality tests (Kolmogorov–Smirnov, Shapiro–Wilk, Anderson-Darling), taking into account their limitations in medium-sized samples; skewness coefficients; visual inspection of histograms and quantile-quantile plots (Q-Q plots).

For categorical variables, absolute and relative frequencies (%) were calculated.

Inferential statistics were performed using the following tests: chi-square test, Fisher’s exact test (to compare nominal variables between the two diagnostic groups); *t*-test for independent samples (to compare normally distributed continuous variables between the two groups); Levene’s test for homogeneity of variances (to test the equality of variances, a necessary criterion for choosing the appropriate variant of the *t*-test); Mann–Whitney U test (for comparing continuous variables with asymmetric distribution between the two groups); Wilcoxon nonparametric test for a single sample (to compare variables with non-normal distribution with clinical reference values).

For the tests used to verify the hypotheses, we calculated the effect size according to Cohen’s d/Cohen’s r coefficient, using the formula r = z/√*n*, to assess the practical significance of the observed differences. The extended classification of effect size [40] is as follows: very small < 0.10, small < 0.3, medium ≈ 0.3–0.5, and large > 0.5.

To control for the type I error generated by performing multiple tests, we applied the Bonferroni correction [41]. This aims to reduce the risk of falsely identifying a significant difference. The method consists of dividing the initial significance threshold (*p* = 0.05) by the total number of tests performed. The significance threshold was adjusted to 0.0125 (0.05/4) for Wilcoxon tests and to 0.005 (0.05/10) for Mann–Whitney U and *t*-tests. However, considering the exploratory nature of the study and the conservative nature of the correction method, the results were interpreted with caution, mentioning both the corrected threshold and the standard threshold.

## 5. Conclusions

The aim of the study was to evaluate inflammatory markers in patients with perception disorders caused by psychiatric pathology (qualitative perception disorders—illusions, hallucinations), given the potential of these markers to influence the understanding of the etiopathogenesis and management of the disease. The main objectives were to obtain an overview of the inflammatory profile characteristic of psychotic disorders and identify possible differences between different diagnostic groups (schizophrenia and acute psychotic disorders).

The study involved 135 patients aged between 18 and 64, most of whom were admitted to a psychiatric ward for acute symptoms; 69 were diagnosed with schizophrenia and 66 with acute psychotic disorder. From a demographic point of view, the two diagnostic groups were relatively homogeneous (most patients were male, from urban areas, with an average level of education and without a stable partner), the only significant difference being the lower average age in the acute psychotic disorder group—a finding that corresponds with the data in the literature. In the schizophrenia group, the predominant clinical form was paranoid schizophrenia, while in the acute psychotic disorder group, acute and transient unspecified psychotic disorder predominated.

At the descriptive statistics level, we identified slightly increased median values compared to the indicative reference values extrapolated from previous studies (for derived indices) or compared to the values established in the guidelines (for CRP) for the NRL, MRL, SII, and CRP markers; in the case of MRL, SII, and CRP, the differences were statistically significant, with a medium (MRL, SII) and large (CRP) effect size, suggesting at least minimal persistent inflammation (low-grade) specific to chronic diseases such as schizophrenia.

Also, at a descriptive level, we observed that the group with acute psychotic disorder had the widest range of values (except for lymphocytes) and the highest maximum values (including extreme values, e.g., CRP 63.4 mg/L), but the highest mean/median values were in the schizophrenia group—consistent with the literature describing intense acute inflammation in acute psychosis and chronic, constant, and moderate inflammation in schizophrenia; potentially statistically significant differences were identified only in CRP (significant according to the standard threshold of *p* = 0.05, but insignificant after applying the Bonferroni correction method, whose purpose is to avoid identifying false significance when performing multiple independent tests—10 in this case, with a significance threshold of *p* = 0.005)—further research is needed to explore these differences more clearly and in greater detail.

In conclusion, the data obtained are largely consistent with those reported in the literature, highlighting the presence of an inflammatory process in psychiatric disorders with perceptual symptoms and the potential of markers to identify inflammation in these disorders. The study makes a relevant contribution by providing a detailed analysis of the differences between the two clinical subgroups, offering additional insight into the understanding of the inflammatory profile specific to schizophrenia and acute psychotic disorder, as well as including markers that have been insufficiently studied in the context of psychiatric pathology, paving the way for future investigations.

Potential future directions for research include longitudinal studies tracking the evolution of markers over time and their relationship to disease phases; investigating causality; expanding the range of markers evaluated to obtain a more complete picture of the inflammatory profile; correlating inflammatory markers with the severity of psychotic symptoms, cognitive functioning, or neuroimaging aspects; and evaluating treatment response based on inflammatory marker values and investigating the potential for treatment personalization.

## Figures and Tables

**Table 1:** Descriptive statistics for numerical variables.

	Mean ± SD/ Median [Q1–Q3]	Minimum	Maximum	Skew
Age	39.5 ± 11.09	18	64	0
Leukocytes	7.26 [6.22–9.02]	3.62	16.99	1.14
Neutrophils	4.22 [3.28–5.75]	1.62	11.65	1.13
Lymphocytes	2.24 ± 0.76	1	5.33	0.96
Monocyte	0.62 [0.5–0.76]	0.24	3.12	3.86
NRL	2.11 [1.41–2.79]	0.6	7.66	1.43
PRL	123.04 ± 47.89	39.69	271.71	0.73
MRL	0.29 [0.23–0.38]	0.1	1.15	2.34
SII	518.84 [322.7–723.18]	146.43	3165.43	2.88
CRP	2.33 [1.07–4.38]	0.3	63.4	4.76
ESR	9 [4–13.25]	2	73	2.9

NLR = neutrophil/lymphocyte ratio; PRL = blood platelet/lymphocyte ratio; MRL = monocyte/lymphocyte ratio; SII = systemic immune–inflammatory index; CRP = C-reactive protein; ESR = erythrocyte sedimentation rate.

**Table 2:** Demographic characteristics by diagnosis.

	Schizophrenia (*n* = 69)	Acute Psychotic Disorder (*n* = 66)
Age (Average ± SD)	43.58 ± 9.59	35.23 ± 11
Sex, *n* (%)		
- Male	63 (91.3%)	64 (96.97%)
- Female	6 (8.7%)	2 (3.03%)
Environment of origin, *n* (%)		
- Urban	61 (88.41%)	52 (78.79%)
- Countryside	8 (11.59%)	14 (21.21%)
Level of education, *n* (%)		
- <9 years	16 (23.19%)	11 (16.67%)
- 9–12 years	39 (56.52%)	40 (60.61%)
- >12 years	14 (20.29%)	15 (22.73%)
Relationship status, *n* (%)		
- Stable partner	20 (28.99%)	28 (42.42%)
- No steady partner	49 (71.01%)	38 (57.58%)

**Table 3:** Inflammatory markers by diagnostic groups.

	Schizophrenia				Acute Psychosis			
	Mean ± SD/Median [Q1–Q3]	Min	Max	Skew	Mean ± SD/Median [Q1–Q3]	Min	Max	Skew
WBC	7.58 [6.57–9.4]	3.65	15.8	1.05	6.94 [6.06–8.6]	3.62	16.99	1.3
NEU	4.41 [3.65–6.34]	1.97	10.14	0.87	3.86 [3.04–5.59]	1.62	11.65	1.42
LYM	2.26 [1.72–2.6]	1	5.33	1.13	2.2 [1.57–2.62]	1.02	4.42	0.76
MON	0.63 [0.5–0.79]	0.24	2.42	3.02	0.62 [0.49–0.71]	0.3	3.12	4.2
NRL	2.25 [1.48–2.97]	0.6	5.5	0.9	1.85 [1.35–2.65]	0.82	7.66	1.81
PRL	124.89 ± 48.16	48.78	241.35	0.78	121.12 ± 47.9	39.69	271.71	0.7
MRL	0.29 [0.23–0.38]	0.12	0.91	1.62	0.28 [0.23–0.38]	0.1	1.15	2.56
SII	528.84 [355.6–724.56]	162.45	2107.91	1.8	465.01 [289.77–712.73]	146.43	3165.43	3.68
CRP	3.3 [1.82–4.38]	0.3	24.4	2.53	1.88 [0.82–3.59]	0.35	63.4	4.28
ESR	10 [6–15]	2	39	1.19	7 [4–12]	2	73	3.56

WBC = white blood cells; NEU = neutrophils; LYM = lymphocytes; MON = monocytes; NRL = neutrophil/lymphocyte ratio; PRL = platelet/lymphocyte ratio; MRL = monocyte/lymphocyte ratio; SII = systemic immune–inflammatory index; CRP = C-reactive protein; ESR = erythrocyte sedimentation rate.

**Table 4:** Wilcoxon test for a single sample.

Marker	Mediana	Normal Upper Limit	W	Z	N	*p*-Value	Interpretation
NRL	2.11	2	5366	1.7	135	0.089	Insignificant
SII	518.84	400	6963	5.21	135	<0.001	Highly significant
CRP	2.33	1	8367	8.3	135	<0.001	Highly significant
MRL	0.29	0.25	6881.5	5.24	134	<0.001	Highly significant

**Table 5:** Mann-Whitney U tests.

	U	z	*p* (Asymptotic)	r	Interpretation
Leukocytes	1928	−1.54	0.124	0.13	Insignificant
Neutrophils	1945	−1.46	0.144	0.13	Insignificant
Lymphocytes	2194	−0.37	0.715	0.03	Insignificant
Monocytes	2094	−0.81	0.42	0.07	Insignificant
NRL	2022	−1.12	0.262	0.1	Insignificant
MRL	2223	−0.24	0.812	0.02	Insignificant
SII	2007	−1.19	0.235	0.1	Insignificant
CRP	1733	−2.4	0.017	0.21	Insignificant according to the corrected threshold (0.005)
ESR	1691	−1.71	0.088	0.15	Insignificant

U = statistical value of the test; z = standardized value of the test; r = effect size.

**Table 6:** *t*-test for independent samples.

	t	df	*p*	Cohen’s d	Interpretation
PRL	0.46	133	0.649	0.08	Insignificant

PRL = ratio of blood platelets to lymphocytes; t = statistical value of the test; df = degrees of freedom; Cohen’s d = effect size.

## Data Availability

Dataset available on request from the authors.
